# Efficiency in Cow-Calf Systems With Different Ages of Cow Culling

**DOI:** 10.3389/fvets.2020.00476

**Published:** 2020-08-05

**Authors:** Amir Gil Sessim, Tamara Esteves de Oliveira, Fredy Andrey López-González, David Santos de Freitas, Júlio Otávio Jardim Barcellos

**Affiliations:** ^1^Department of Animal Science, Faculty of Agronomy, Núcleo de Estudos em Sistemas de Produção de Bovinos de Corte e Cadeia Produtiva (NESPro), Federal University of Rio Grande do Sul (UFRGS), Porto Alegre, Brazil; ^2^Plant Ecology Laboratory, University of Vale do Rio dos Sinos (UNISINOS), São Leopoldo, Brazil

**Keywords:** beef cattle, British breeding, feed restriction in cows, herd structure, longevity, stayability

## Abstract

The bioeconomic efficiency of cow-calf systems was compared by a deterministic dynamic simulation. The simulation model considered stable cow-calf systems differentiated by the maximum age for culling cows, lifetime, culled at 4–13 years old. The necessary supply of metabolizable energy for the herd was established as natural grasslands, cultivated pasture in the winter/spring, and pre-dried pasture produced by the system. The biological efficiency of the systems was considered the ratio between the production of total live weight and the metabolizable energy consumed over one production cycle. Economic efficiency was determined by the ratio between gross margin and production area and the ratio between gross margin and number of cows. Bioeconomic efficiency was determined by a simple linear regression between biological efficiency, economic efficiency per area, and economic efficiency per cow. The efficiency of the animal unit, considering biological efficiency and economic efficiency per area were better in the system that culled cows at 4 years old, while economic efficiency per cow was better in the system that culled cows at 13 years old. In determining the bioeconomic efficiency of the systems, the best results were found in the system that culled cows at 6 years old, which suggests that the best efficiency of a cow-calf herd is reached when the adult age and mature weight of the cow are reached, and there is no more energy used for growing. The results indicate that stable cow-calf herds express their best biological efficiency and economic efficiency per area when the cow culling age is lower. However, economic efficiency per cow depends on cows that remain in the herd as long as possible. The culling age of cow that balances these biological and economic indicators is reached around 5 and half years.

## Highlights

- Simulation model able to predict the ideal age of cow culling for the best bioeconomic efficiency of cow-calf systems in different environments and markets.
- The younger the cow culling, the greater the bioeconomic efficiency per unit of production area.
- The older age at culling allows a greater economic efficiency per cow, even with lower biological efficiency.
- Production scenarios that allow intensification of cultivated pastures along with markets that value culling cows are indicated for herds that cull young cows.
- Regions where the intensification is not viable, and the calf is the most valued product should be prioritized by systems that cull older cows.

## Introduction

The economic viability of cow-calf systems is associated with many factors such as environmental, socio-economic, labor, biological efficiency of cows, and others. Therefore, to achieve satisfactory results in the activity, the herd must be structured to reach the highest possible productive capacity of these animals when transforming feed resources into a commercialized product. With this purpose, research has measured the biological efficiency of the cow-calf system through the use of metabolizable energy (ME) for calf production (1, 2). However, these studies evaluated the individual efficiency of cows and did not consider the efficiency of the cow-calf systems, which can distort the results of these analyzes.

When analyzing ME, the impact of the metabolizable energy for the maintenance (ME_m_) of the production systems must be considered, which is the unproductive portion of the ME and represents about 50% of the total herd requirement only for cow maintenance (3). This is because, the older the animals, the more energy they need for maintenance until they reach their mature weight (4), which makes them less efficient in transforming the energy consumed into muscle, hence, meat products. Although the aim of farmers is simpler, producing a calf per cow/year, the conversion ratios of ME into live weight kilogram is what determine the efficiency of cow-calf system. This difference in the use of ME makes this indicator relevant for efficiency analysis of production systems, as well as the economic impact of the variation in the culling age of cows.

When considering cow-calf systems, the shorter lifetime cow in the herd demands high heifer replacement, which allows better utilization of the energy used for growth (5, 6). In addition, cows that reach mature weight consume about 25% more energy for maintenance than 2-year-old cows (4). On the other hand, when the productive lifetime of cows is longer, heavier calves are obtained at weaning, and there is a reduction in the heifer replacement rate (7), which positively impacts the margin of the cow-calf system. Thus, understanding these relations, especially considering production systems, allows farmers to assess which is the best strategy to increase the productivity of their operations.

In this context, in production systems, the herd structure is a complex issue and must consider the ideal age for cow culling (lifetime). Although some research indicates that the most efficient systems culled primiparous cows soon after weaning a calf (8–10), this system could not be maintained because it is not able to produce the necessary number of heifers to replace the culled cows and ensure herd stability (6). Nevertheless, this hypothesis must be scientifically validated, considering the different herd structures and bioeconomic efficiency to identify the ideal cow lifetime in the herd.

In addition, research that seeks to increase animal production efficiency contributes to the strategic use of natural resources and helps both to reduce the negative impact of production on the environment (11) and to the image of beef industry. However, comparison through experimentation becomes impracticable due to the cost, complexity, and level of control required by these systems for a reliable analysis. In contrast, the simulation models analyze the interaction between the production system factors quickly and at low cost (12). In this sense, this study identifies the ideal cow lifetime in the herd until its culling for the best bioeconomic efficiency of cow-calf systems.

## Methods

### Model Overview

A deterministic dynamic model was constructed to compare the bioeconomic efficiency of cow-calf systems programmed in Microsoft Excel spreadsheets. The input parameters were collected from 18 scientific manuscript published in relevant journals, three systems of nutritional requirements, as well as the technical coefficients and assumptions of herd evolution typical of cow-calf systems in natural grasslands (13).

Ten production system scenarios were built with herds of 1,000 Aberdeen Angus cows. The criteria for differentiating the systems was the maximum age at which the cows were culled, called lifetime (LT), considered at 4 (LT4); 5 (LT5); 6 (LT6); 7 (LT7); 8 (LT8); 9 (LT9); 10 (LT10); 11 (LT11); 12 (LT12); and 13 years old (LT13). To maintain the discrete nature of the variables, the maximum age for culling cows in the system was considered after the weaning of its last calf (e.g., 10.5-year-old cows for the LT11).

To compare the systems, the following parameters were used: (1) stable herd structure, with the distribution effect of the age groups of cows and bulls based on zootechnical indicators (Table 1); (2) body condition score 3 on a scale of 1 (very thin) to 5 (very fat) (25); (3) mineral salt offer in the order of 80 g/450 kg of live weight (LW) per day for all cows; 4. Vaccination against clostridiosis, reproductive diseases, foot-and-mouth disease, brucellosis, in addition, antiparasitic treatments, according to typical proceedings, and legal requirements for animal welfare and sanitation.

**Table 1:** Assumptions of simulation model for the efficiency of cow-calf systems according to lifetime cows.

**Model inputs**	**Value**	**References**
**Mature cow weight at 5 years (kg)**	480.0	
**Calving rate (%)**		
2-year-old cows	88	
3-year-old cows	87	(7, 14)
Cows above 3 years old	88	
**Birth weight (kg)**		
Calf of 2-year-old cow	35.0	
Calf of 3-year-old cow	36.1	(15)
Calf of 4-year-old cow	37.2	
Calf of cow above 4 years	38.0	(4)
**Peak milk production (kg)**		
2-year-old cows	5.92	(16–20)
3-year-old cows	7.04	
Cows above 3 years old	8.00	
**Average daily gain (ADG)(kg)**		
Calf of 2-year-old cow	0.633	(7, 14, 15, 21)
Calf of 3-year-old cow	0.656	
Calf of 4-year-old cow	0.703	
Calf of 5 to 9-year-old cow	0.770	
Calf of 10-year-old cow	0.763	
Calf of 11-year-old cow	0.753	
Calf of 12 and 13-year-old cow	0.733	
From weaning to 12 months	0.800	(4)
1-year-old cows (13–24 month)	0.267	(22)
2-year-old cows	0.160	
3 and 4-year-old cows	0.053	(23)
11-year-old cows	−0.008	(7)
12 and 13-year-old cows	−0.009	
**Mortality rate (%)**		
Calf of 2-year-old cow	5	(24)
Calf of 3-year-old cow	2	
From weaning to 12 months	3	
Cows	2	

**Figure 1:**
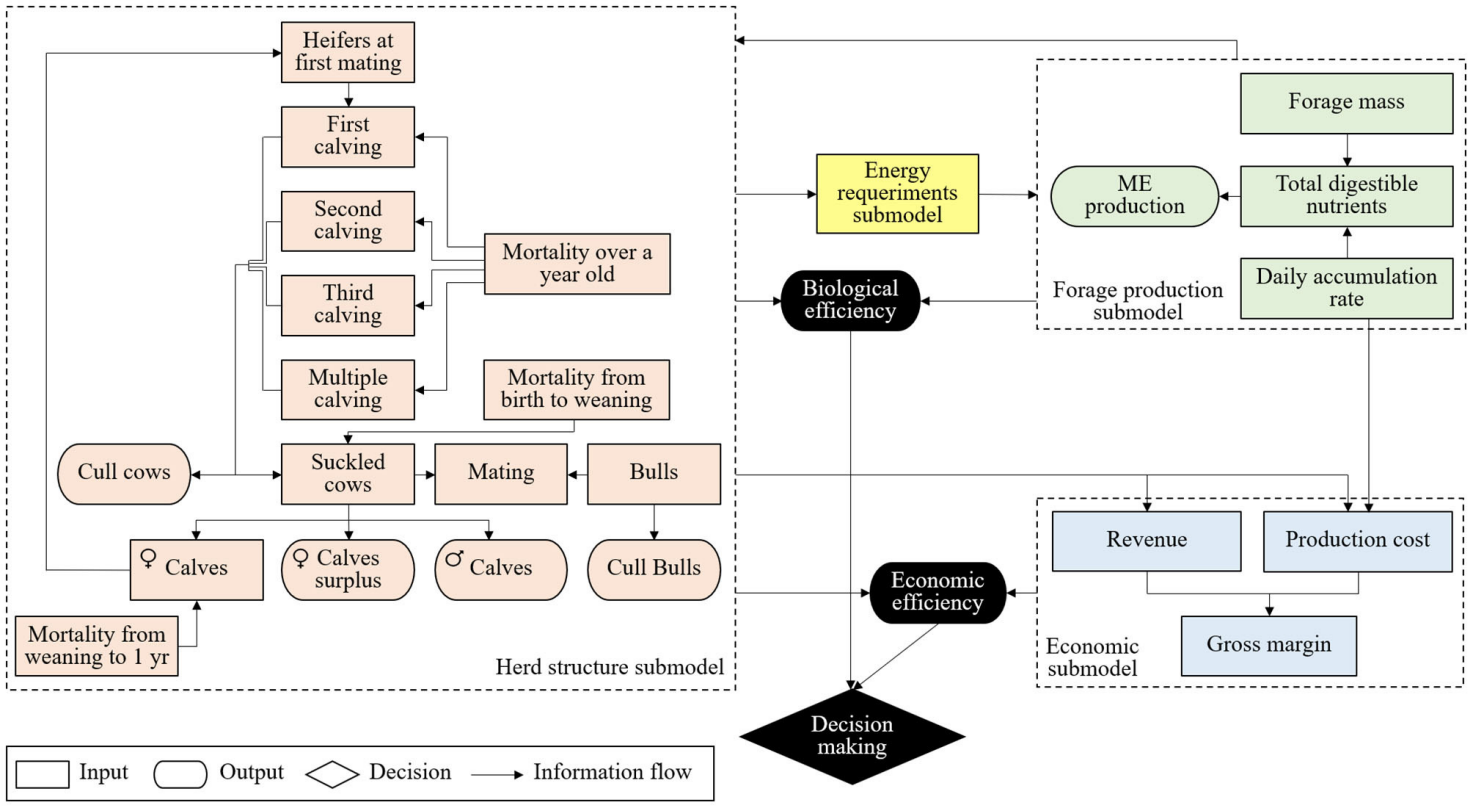
Simplified flowchart of the proposed conceptual model for cow-calf systems with different lifetime cow.

The model was developed by the interaction between the dynamics of cow-calf herd structure, animal energy necessity, energy production by grasslands, and monetary flow of the production system (24, 26). For this, the submodels were developed: herd structure, energy requirement, forage production, and economic (Figure 1).

### Herd Structure Submodel

The breeding season was considered from November 1st to January 29th (90 days). The initial mating age considered for heifers was 14 months and for bulls at 2 years. In this model, bulls were purchased at 2 years, used for six breeding seasons until 7 years old, and subsequently sold with an annual culling rate of 20%. Cows at 2 years or more were exposed to natural breeding under the ratio of one bull for every 25 females. It has been established that heifers were artificially inseminated (A.I.) at 14 months with the synchronization program based on prostaglandin (PGF2a) administrations, considering 1.6 A.I. by heifers (27, 28). The pregnancy rate considered at 30 days after insemination was 92% and the fetal mortality rate between the 30th and full term was 7% (29, 30). The calving season was divided into four periods of 21 days each, from August 16th to November 18th (24).

**Figure 2:**
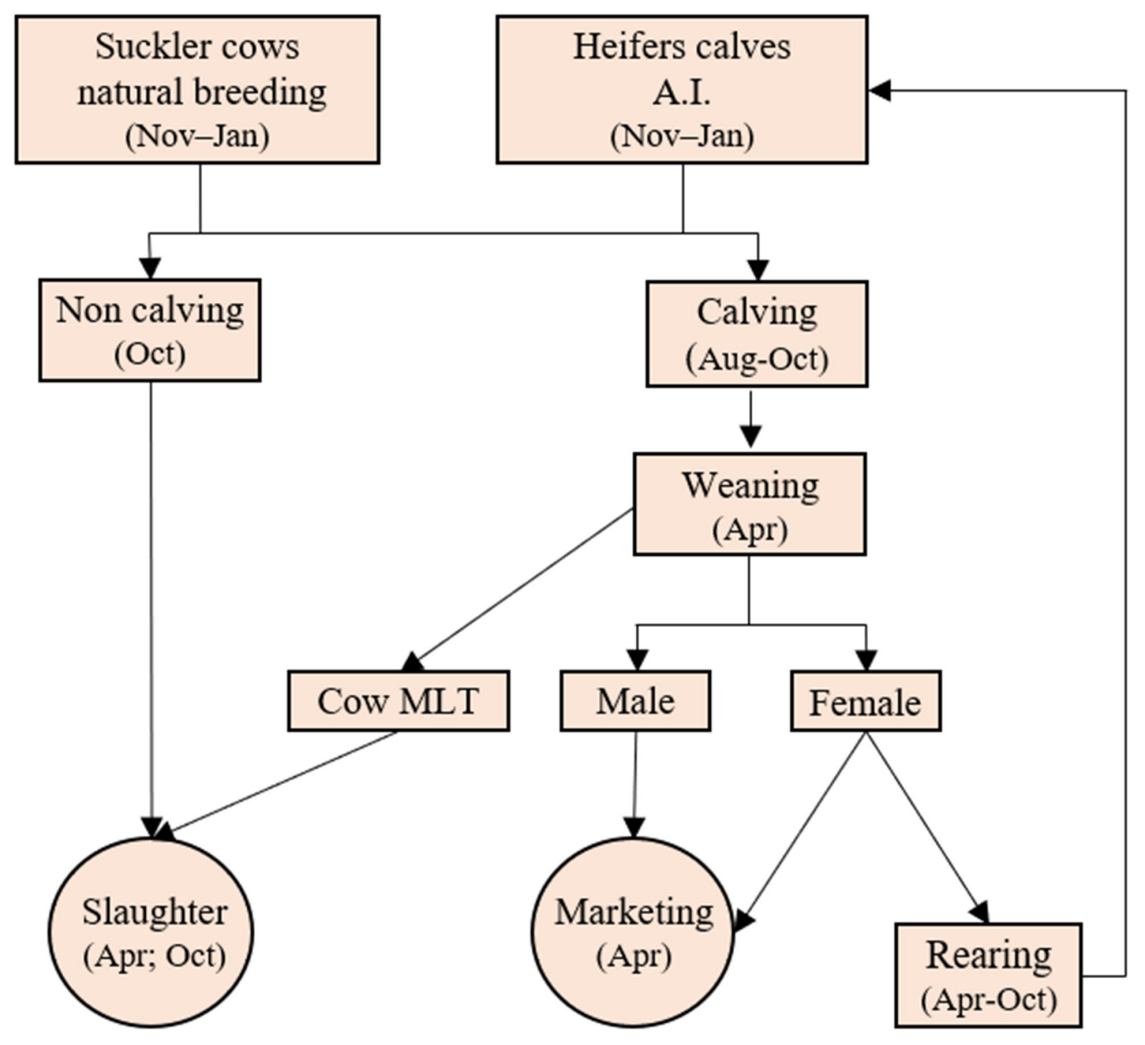
Cow-calf system flowchart (A.I., artificial insemination; MLT, maximum lifetime).

The proportion of cows in each age group varied according to the maximum age of culling and the calving, weaning, and mortality rates. Cows that did not calve a calf were culled at the end of calving season. Those that did not wean their calves or reached the arbitrated LT for each system were culled at weaning on April 1 (Figure 2). The calving and weaning rates were represented by the number of cows that calved and weaned a calf, respectively, in relation to the number of cows subjected to mating in the previous season.

The heifer replacement considered only those produced in the system itself to keep the herd with a constant number of cows, without external purchases. When the number of heifers produced did not reach the minimum necessary for replacement, the system was not simulated, since it would not remain sustainable. For a 3-year LT system, a heifer replacement rate of 52% would be required, which would be possible only with sexed semen in artificial insemination to increase its weaning rates for female calves (6). For this reason, LT4 was the youngest LT simulated, as culling cows under 4 years old would cause a gradual reduction of the system until it became unviable. Heifer retention was performed at weaning, and the selection of the heaviest heifer was used as a criterion.

Female calves that did not recompose the system and male calves were sold immediately after weaning. The total production (TP) of the scenarios was calculated from the sum of all of the kg sold in live weight of each category, while the productivity (kg/ha) was determined from the relation between TP and the production area, as described by Nasca et al. (31). For all the systems, the same weight was considered for animals in the same category that were sold and that remain in the herd. For instance, if a 4-year-old cow weighing 460 kg was sold, those of the same age that remained in the system also weighed 460 kg at that time.

### Energy Requirements Submodel

The energy requirement submodel evaluated the daily metabolizable energy (ME) needs for calves, cows, and bulls, considering the age group and the physiological state in which each animal is in the productive cycle; maintenance (ME_m_), growth (ME_g_), lactation (ME_l_), pregnancy (ME_y_); according to the previously defined calving periods. The modeling considered the availability of 100% of the energy necessary for the animals to reach the productive, individual, and herd indexes, according to the dynamic physiological parameters of each animal age group (4, 21, 32). Biological efficiency, by age group of the cow (BioEV_v_), was defined by Equation (1).

(1) $$\begin{array}{l} BioEC_{c}=MECC_{c}/\left(WGC_{c}+\;LWCWC_{c}\right) \end{array}$$

in which, BioEC_c_ is the biological efficiency of the cow; MECC_c_ is the total metabolizable energy consumed by the cow; WGC_c_ is the weight gain of the cow; LWCWC_c_ is the live weight of the cow's weaned calf; c is the age group of the cow.

The BioE (Equation 2) of the different simulated systems was also evaluated from the relationship between the total ME consumed by the system and the kg sold of slaughtered bulls and cows, in addition to the kg of calves (1, 2).

(2) $$\begin{array}{l} BioE_{t}=MEC_{t}/TLWS_{t} \end{array}$$

In which, BioE is the biological efficiency of the system; MEC is the total metabolizable energy consumed by the system; and TLWS is the total live weight sold; t is the maximum lifetime cow (LT).

### Forage Production Submodel

This submodel was used to calculate the stocking rate capacity of each cow-calf system. The metabolizable energy of forages (MEF; Equation 3) is estimated from forage production, through the daily accumulation rate (DAR), forage mass (FM), grazing rate, and total digestible nutrients (TDN).

(3) $$\begin{array}{l} MEF=TDN\;\times \;4.4\;\times \;0.82 \end{array}$$

in which MEF is the metabolizable energy of forage; TDN is the total digestible nutrient; 4.4 is the conversion constant from TDN to digestible energy; and 0.82 is the conversion constant of digestible energy for ME (4).

Thus, the model simulates the monthly variation in ME production in response to the demand for ME from animals in the herd. Therefore, the model was built to adjust the sizing of the production area with the assumption that in the months with feed surplus there would be storage through pasture conservation, in the form of pre-dried, to be supplied in periods of feed deficit. It was assumed as a feed surplus when the pasture produced more ME than the animals required (May to August). The feed deficit was considered when pasture production did not have the capacity to supply animals' ME necessity (September to March). Forage storage was considered only for cultivated oat pastures (*Avena strigosa*) in consortium with ryegrass (*Lolium multiflorum*), as the scarcity of natural grasslands surplus and the high cost of this hay discourage the practice in the region.

The values for the calculation of ME production used to build this model were based on data from natural grasslands typical of the region (24) and from scientific research on cultivated oat/ryegrass pastures (33–35) from FM and DAR in the range of two standard deviations from the mean. While natural grasslands were used over the year, oat pasture (April to August) with ryegrass (May to October) was used from April to October.

To determine which age groups of calves should receive natural grassland or oats/ryegrass, the daily dry matter intake capacity (DMI_d_) was considered in the different physiological conditions of the animals (32). To calculate the metabolizable energy consumed per day (MEC_d_), Equation (4) was used:

(4) $$\begin{array}{l} MEC_{t}=DMI_{t}\;\times \;MEF \end{array}$$

in which, MEC_d_ is the metabolizable energy consumed per day; DMI_t_ is the daily dry matter intake; and MEF is the metabolizable energy of forage; t is the number of days.

The available metabolizable energy (AME) per hectare was determined from Equation (5):

(5) $$\begin{array}{l} AME=EMF\;\times \;\left(MF+TAD_{t}\right)\;\times \;ECF \end{array}$$

in which, AME is the metabolizable energy available per hectare; MEF is the metabolizable energy of forage; FM is the forage mass per hectare; DAR is the daily accumulation rate (36); t is the number of days; and EHF is the efficiency of harvest forage used by the animals (37).

**Figure 3:**
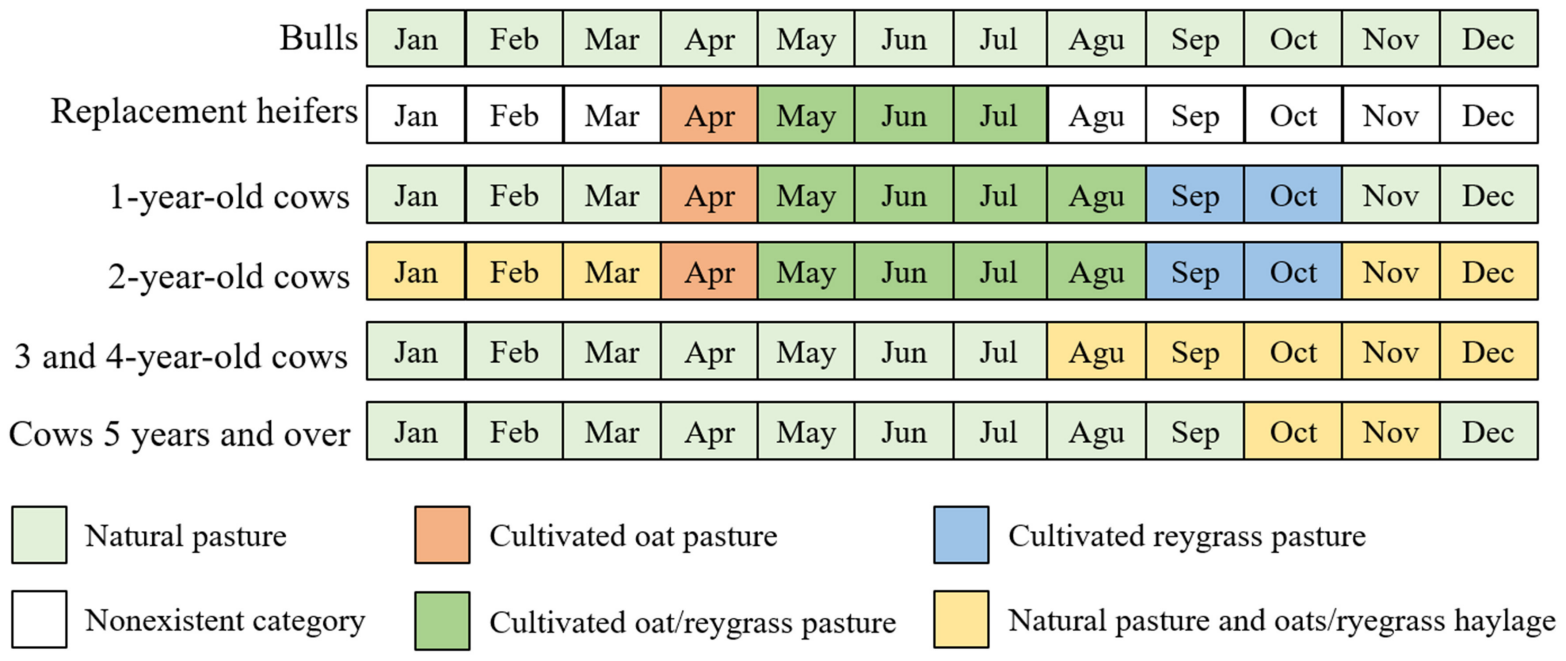
Monthly feed composition provided for each age group of cows and category of cow-calf systems.

Thus, the average AME of natural grasslands, in 12 months of the year, and of oat/ryegrass consortium pasture, in 7 months of production, obtained average values of 364 Mcal/ha and 1,744 Mcal/ha, respectively. Considering the energy requirements of the systems and the energy availability of the pastures (Figure 3), the feed base of each category and age group of animals in the different systems was determined.

### Economic Submodel

The economic submodel measured the economic efficiency per area (EEA) and per cow (EEC) in the different scenarios, through the gross margin (GM), which was calculated by the difference between revenue and production cost (fixed and variable) and later divided by the number of hectares and the number of cows exposed to breeding in the previous year (Equations 6, 7).

(6) $$\begin{array}{lll} EEA & = & GM/hectares \end{array}$$

(7) $$\begin{array}{l} EEC=GM/number\;of\;cows\;exposed\;to\;breeding\\ \;in\;the\;previous\;year \end{array}$$

Total revenue was estimated by the sum of the total sale of LW kg of weaned male calves, weaned female calves not used for replacement, and culled cows and bulls. The prices were based on the regional average of the last 5 years (38, 39) that also agrees with the current market conditions in the country. Therefore, the selling price used per kg of LW for male and female calves was US$ 1.58 and US$ 1.45, respectively, for cows sold at the end of calving US$ 1.15, for cows sold at the end of weaning US$ 1.08, and for cull bulls US$ 1.15.

The fixed costs considered in the model were those that do not change with the variation of production in the cow-calf system in a productive cycle, while the variable costs were those that show variability in the increase or decrease in production. All costs were considered in accordance with market prices and subsequently corrected by the General Price Index-Internal Availability (í*ndice geral de preços-disponibilidade interna—IGP-DI*) for the average of the last 5 years. The values used are representative of a property of 1,000 cows located in southern Brazil with the tax values varying proportionally to the production area. The model did not consider opportunity costs for land and capital, as they are not parameters usually used by farmers in Brazil.

To evaluate the relation between BioE and EEA and between BioE and EEC, simple linear regression models were performed using the SPSS 20.0 software (40), considering a significance level of 95%. The adjusted trend lines were plotted on a dual-axis graph to determine the balance between the two efficiencies. Hence, it was possible to identify the lifetime cow in the herd that resulted in the best bioeconomic efficiency.

For all models, a manual check of the input parameters and the results obtained were performed to detect distortions and possible typing errors. In addition, careful validation based on several scientific references (1, 3, 6, 7, 12, 24, 41–43) was carried out to ensure the model's representativeness.

## Results

### Herd Structure Submodel

The herd composition, the number of heifers retained for replacement, and the number of calves weaned varied in the different cow-calf systems according to each LT (Table 2). The results showed that the reduction in the culling age cow requires a higher replacement of heifers. The LT4 obtained a higher replacement of heifers than LT6 (47.7%), LT8 (83.3%), and LT13 (145.5%).

**Table 2:** Herd structure (%) of 10 cow-calf systems (1,000 cows) with different lifetime cow (LT).

**Category**	**LT4**	**LT5**	**LT6**	**LT7**	**LT8**	**LT9**	**LT10**	**LT11**	**LT12**	**LT13**
2-year-old bulls	0.19	0.22	0.24	0.25	0.26	0.26	0.27	0.27	0.28	0.28
3-year-old bulls	0.19	0.22	0.24	0.25	0.26	0.26	0.27	0.27	0.28	0.28
4-year-old bulls	0.19	0.22	0.24	0.25	0.26	0.26	0.27	0.27	0.28	0.28
5-year-old bulls	0.19	0.22	0.24	0.25	0.26	0.26	0.27	0.27	0.28	0.28
6-year-old bulls	0.19	0.22	0.24	0.25	0.26	0.26	0.27	0.27	0.28	0.28
1-year-old cows	17.02	14.02	12.12	10.85	9.92	9.23	8.70	8.28	7.94	7.66
2-year-old cows	14.98	12.33	10.68	9.55	8.73	8.12	7.65	7.28	6.98	6.74
3-year-old cows	13.03	10.72	9.29	8.30	7.60	7.07	6.66	6.34	6.08	5.87
4-year-old cows	–	9.44	8.17	7.31	6.69	6.23	5.86	5.58	5.35	5.16
5-year-old cows	–	–	7.18	6.43	5.88	5.47	5.16	4.91	4.71	4.54
6-year-old cows	–	–	–	5.66	5.18	4.82	4.54	4.32	4.13	4.00
7-year-old cows	–	–	–	–	4.56	4.24	3.99	3.80	3.64	3.52
8-year-old cows	–	–	–	–	–	3.74	3.51	3.34	3.21	3.10
9-year-old cows	–	–	–	–	–	–	3.09	2.94	2.81	2.72
10-year-old cows	–	–	–	–	–	–	–	2.59	2.48	2.40
11-year-old cows	–	–	–	–	–	–	–	–	2.19	2.10
12-year-old cows	–	–	–	–	–	–	–	–	–	1.86
Cull bulls	0.19	0.22	0.24	0.25	0.26	0.26	0.27	0.27	0.28	0.28
Cull cows	16.12	13.09	11.18	9.88	8.94	8.25	7.71	7.29	6.95	6.67
Replacement heifers	17.02	14.02	12.12	10.85	9.92	9.23	8.70	8.28	7.94	7.66
Male calves	19.15	19.84	20.28	20.57	20.79	20.95	21.07	21.17	21.25	21.31
Female calves	1.55	5.22	7.54	9.10	10.23	11.09	11.74	12.26	12.66	13.01
Total	100	100	100	100	100	100	100	100	100	100

The systems also varied concerning the weaning rate, the number of animals sold, the average weight per category, the total kg sold, and their distribution by category (Table 3). Systems that culled older cows had a higher rate and weight at weaning than those that culled younger cows. However, production per animal was higher in the younger culled cow systems.

**Table 3:** Number and average weight per animal sold and weight of calves at weaning in 10 cow-calf systems with different lifetime cow (LT).

**Systems**	**Bulls**	**Cows**	**Male calves**	**Female calves**	**Total**
	**Head (Kg/hd)**	**Head (Kg/hd)**	**Head (Kg/hd)**	**Head (Kg/hd)**	**Head (Kg/hd)**
LT4	5 (768)	358 (457)	425 (185)	34 (150)	821 (306)
LT5	5 (768)	281 (463)	427 (190)	112 (163)	825 (283)
LT6	6 (768)	236 (462)	427 (192)	159 (167)	828 (268)
LT7	6 (768)	206 (462)	428 (194)	189 (173)	829 (260)
LT8	6 (768)	184 (462)	428 (195)	211 (177)	829 (254)
LT9	6 (768)	169 (462)	428 (196)	227 (180)	830 (250)
LT10	7 (768)	157 (462)	429 (197)	239 (181)	832 (246)
LT11	7 (768)	148 (461)	429 (197)	248 (182)	832 (244)
LT12	7 (768)	140 (460)	429 (197)	256 (183)	832 (242)
LT13	7 (768)	134 (459)	429 (197)	262 (183)	832 (240)

*The weaning weight is represented by the average weight of male calves sold. Kg/hd, kilogram/head*.

Systems that remained with their cows for longer, sold more male and female calves and with a higher average weight in both categories. In contrast, herds that culled younger cows, sold lighter cows, but in higher quantities. In addition, the younger the culled cow was, the higher the TP was, even with the lowest number of animals sold. The LT4 showed a 7.1% superiority in kg sold over LT5 due to the higher number of cows sold by age limit. This was the biggest difference between sequential scenarios, similar to the variation found between LT7 and LT13.

### Energy Requirements Submodel

The largest MECC was found in 4-year-old cows and the lowest in 2-year-old cows. Mature weight cows were the ones that used more ME for maintenance functions (74%), while 2-year-old cows the ones used less (66%). In contrast, 2-year-old cows demanded a higher proportion of ME_g_ (14%) than other cows in the herd.

**Figure 4:**
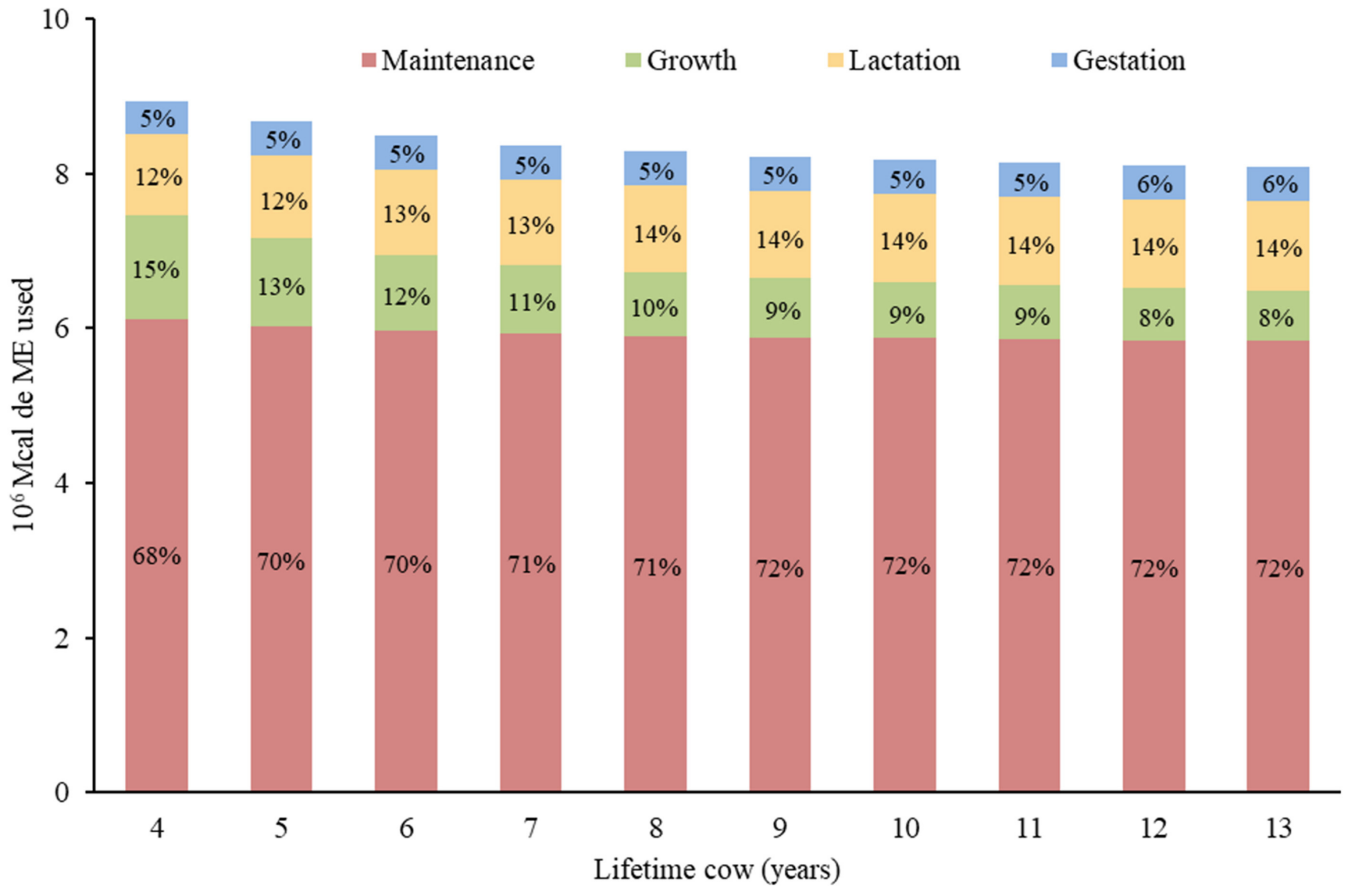
Total metabolizable energy (ME) used in 10 cow-calf systems with different lifetime cow (years) and their respective distribution of ME for maintenance, growth, lactation, and gestation. The numbers in % (middle of the columns) represent the portion destined for each function of the total energy consumed by the system itself.

In relation to the systems, the MEC was higher in younger cows, with a 10.8% superiority of LT4 over LT13. However, systems that remained with 11 to 13-year-old cows did not vary in relation to the use of MEC. Although the total volume of ME_m_ used by LT4 was 4.7% higher than LT13, this energy presented a greater proportion in LT13 (Figure 4). However, for ME_g_, it was not the same, as the LT4 consumed 108.4% more of this energy than the LT13 and was the one that presented the highest proportion of consumption among the systems.

**Figure 5:**
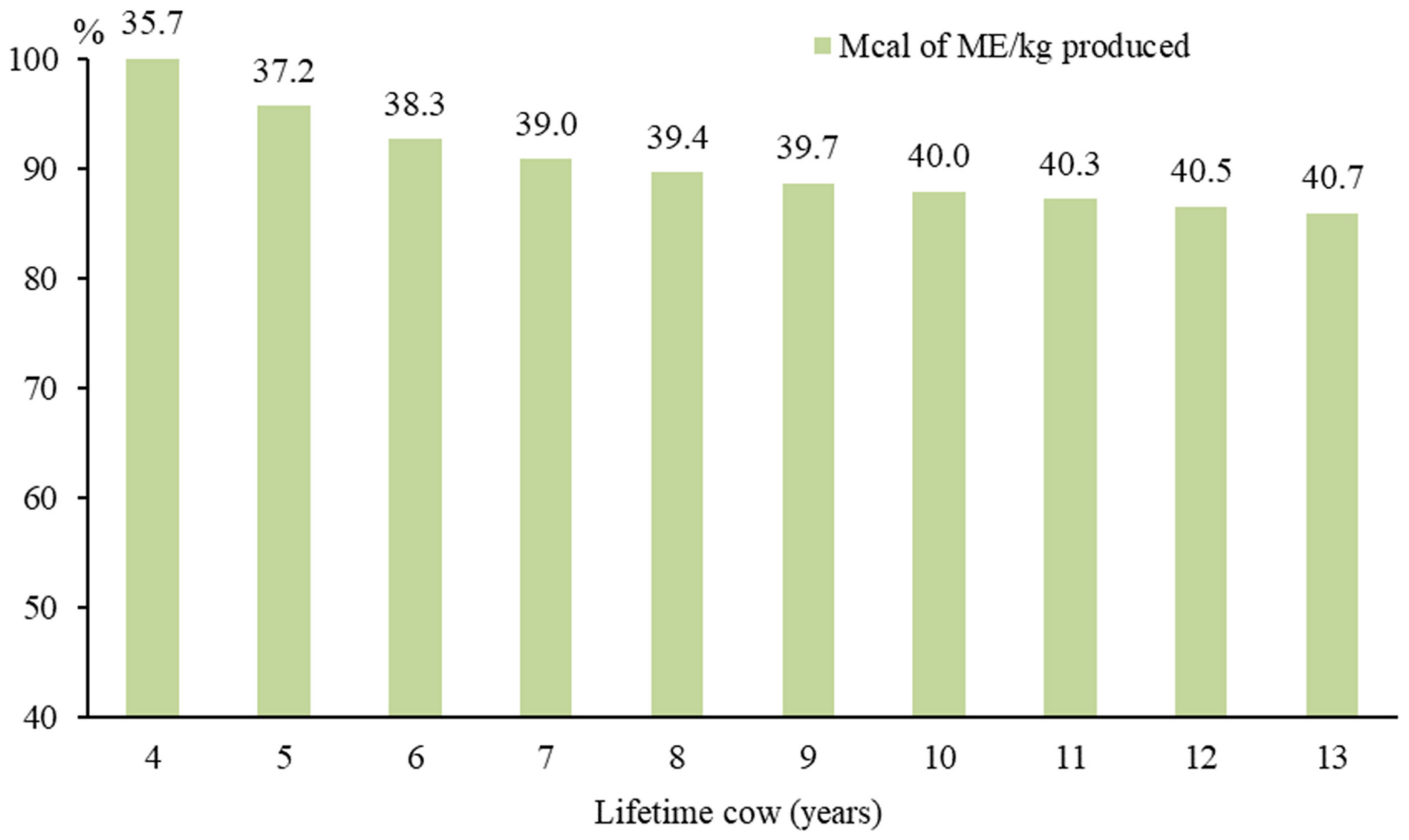
Biological efficiency: Mcal of metabolizable energy (ME; values over the bars) consumed per kg produced represent in 10 cow-calf system with different lifetime cow (LT). The LT4 is the most efficient among all LT, therefore it was represented with 100%.

The BioEC was 21.6% higher in 2-year-old cows than 12-year-old cows, as they needed 33.5 Mcal of ME to produce one kg of LW while the older 40.8 Mcal of ME. In systems, the best BioE was LT4 with a 12.3% superiority over LT13, due to the need for 35.7 Mcal of ME to produce one kg of LW in the smallest LT and 40.7 Mcal of ME in the largest (Figure 5). The LT11 presented BioE only 1% higher than LT13, which demonstrates the proximity between the systems and the formation of a plateau when the cows are culled after 10 years old.

### Forage Production Submodel

Systems that culled younger cows required less production area and had a higher proportion of cultivated pasture compared to systems that culled older cows (Table 4). The LT4 used a production area 23% smaller than LT13, but it needed a 144% larger area of oat/ryegrass pasture.

**Table 4:** Total production area, oat/ryegrass pasture, and productivity in 10 cow-calf systems with different lifetime cow (LT).

	**LT4**	**LT5**	**LT6**	**LT7**	**LT8**	**LT9**	**LT10**	**LT11**	**LT12**	**LT13**
Total production area (ha)	1,210	1,307	1,363	1,400	1,427	1,446	1,461	1,472	1,481	1,489
Oat/ryegrass pasture (%)	29.30	21.73	17.67	15.18	13.50	12.30	11.41	10.73	10.19	9.76
Productivity (kg/ha)	207	178	163	153	147	143	140	137	135	133

As the culling age of cows was reduced, there was an increase in stocking and productivity of the systems. The LT4 obtained higher stocking and productivity of 28 and 55.6%, respectively, in relation to LT13. Ergo, the older the cows were culled, the smaller was the difference between the sequential systems of only 1% from LT11.

### Economic Submodel

**Figure 6:**
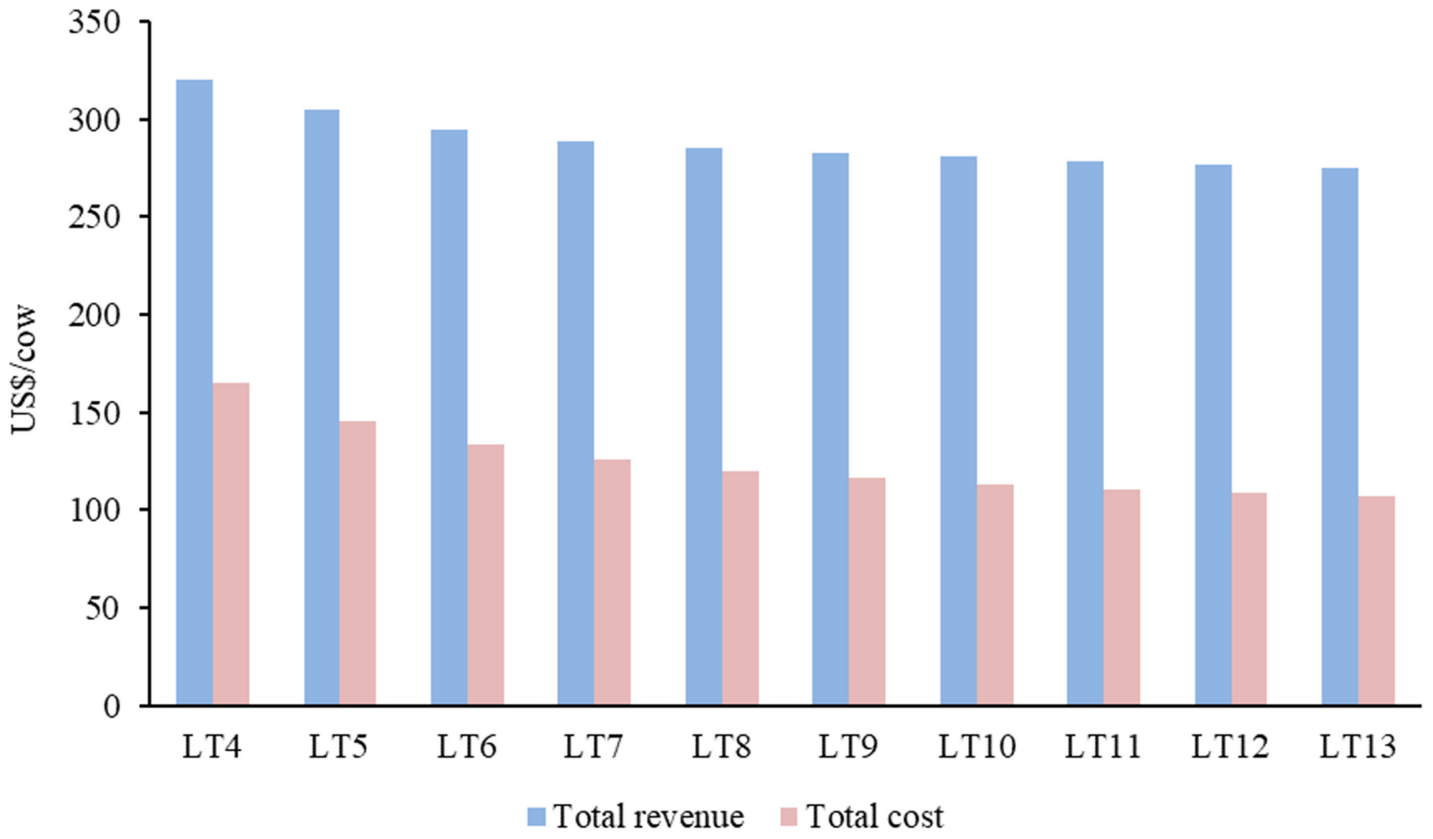
Total revenue (TR) and total cost (TC) per cow in 10 cow-calf systems with different lifetime cow (LT; years).

The lower the LT, the higher was the total revenue (TR) of the cow-calf system (Figure 6). The systems of cows culled older showed the composition of their TR predominantly by male and female calves, representing 73.6% in LT13 (Table 5). In contrast, systems with younger culled cows showed greater participation of cows and bulls in the TR, with LT4 reaching 59%. Despite the increase in TR with the reduction in the culling age of cows, the production costs increased considerably, mainly for feed, which caused a decrease in GM.

**Table 5:** Composition of revenue and costs of production and gross margin of 10 cow-calf systems with different lifetime cow (LT).

**Item**	**LT4**	**LT5**	**LT6**	**LT7**	**LT8**	**LT9**	**LT10**	**LT11**	**LT12**	**LT13**
**Revenues (%)**										
Bulls	1.14	1.35	1.49	1.58	1.64	1.69	1.72	1.76	1.78	1.80
Cows	57.82	48.06	41.43	36.69	33.19	30.55	28.52	26.92	25.61	24.55
Male calves	38.69	41.89	44.00	45.32	46.20	46.87	47.38	47.82	48.17	48.46
Female calves	2.34	8.70	13.08	16.41	18.97	20.89	22.38	23.51	24.44	25.18
Total	100	100	100	100	100	100	100	100	100	100
**Costs (%)**										
**Fixed**										
Accounting	0.14	0.16	0.17	0.18	0.19	0.20	0.20	0.21	0.21	0.21
Electricity	0.58	0.66	0.72	0.77	0.80	0.83	0.85	0.87	0.89	0.90
Taxes	2.41	2.94	3.35	3.65	3.89	4.08	4.24	4.36	4.47	4.56
Maintenance	1.02	1.17	1.29	1.37	1.44	1.50	1.54	1.58	1.61	1.63
Labor	12.16	13.76	14.99	15.92	16.65	17.24	17.71	18.10	18.42	18.69
Insurance	0.62	0.70	0.76	0.81	0.85	0.88	0.90	0.92	0.94	0.95
**Variables**										
Purchase bulls	5.01	6.38	7.40	8.18	8.78	9.27	9.66	9.99	10.26	10.48
Oat/ryegrass	50.54	45.81	42.33	39.68	37.60	35.95	34.61	33.52	32.61	31.85
Fuel^*^	2.86	2.59	2.39	2.24	2.13	2.03	1.96	1.90	1.84	1.80
Veterinarian^**^	0.93	0.84	0.77	0.72	0.69	0.66	0.63	0.61	0.59	0.58
Pre-dried	3.59	3.70	3.63	3.58	3.55	3.52	3.49	3.47	3.46	3.44
Reproduction	3.44	3.14	2.93	2.77	2.64	2.54	2.46	2.39	2.34	2.29
Mineral salt	13.05	14.22	15.12	15.81	16.34	16.76	17.10	17.38	17.61	17.80
Animal health	3.67	3.94	4.16	4.32	4.45	4.55	4.63	4.70	4.75	4.80
Total	100	100	100	100	100	100	100	100	100	100

*Costs represented in US$*.

* *Fuel costs were measured for maintaining cultivated pasture*.

** *The disbursement to the veterinarian was calculated as a cost of cesarean section on 5% of calving from females from 22 to 24 months of age (44)*.

The cost of LT4 was higher than LT8 (37%) and LT13 (53.8%). The greater participation in production costs in all systems was related to the production of cultivated oat/ryegrass pasture for younger females. In addition to pasture, the items that impacted the costs in LT4 to LT6 the most were mineral salt, labor, and the purchase of bulls, presented in order of importance. From the LT7, labor became the second-highest cost and mineral salt the third.

**Figure 7:**
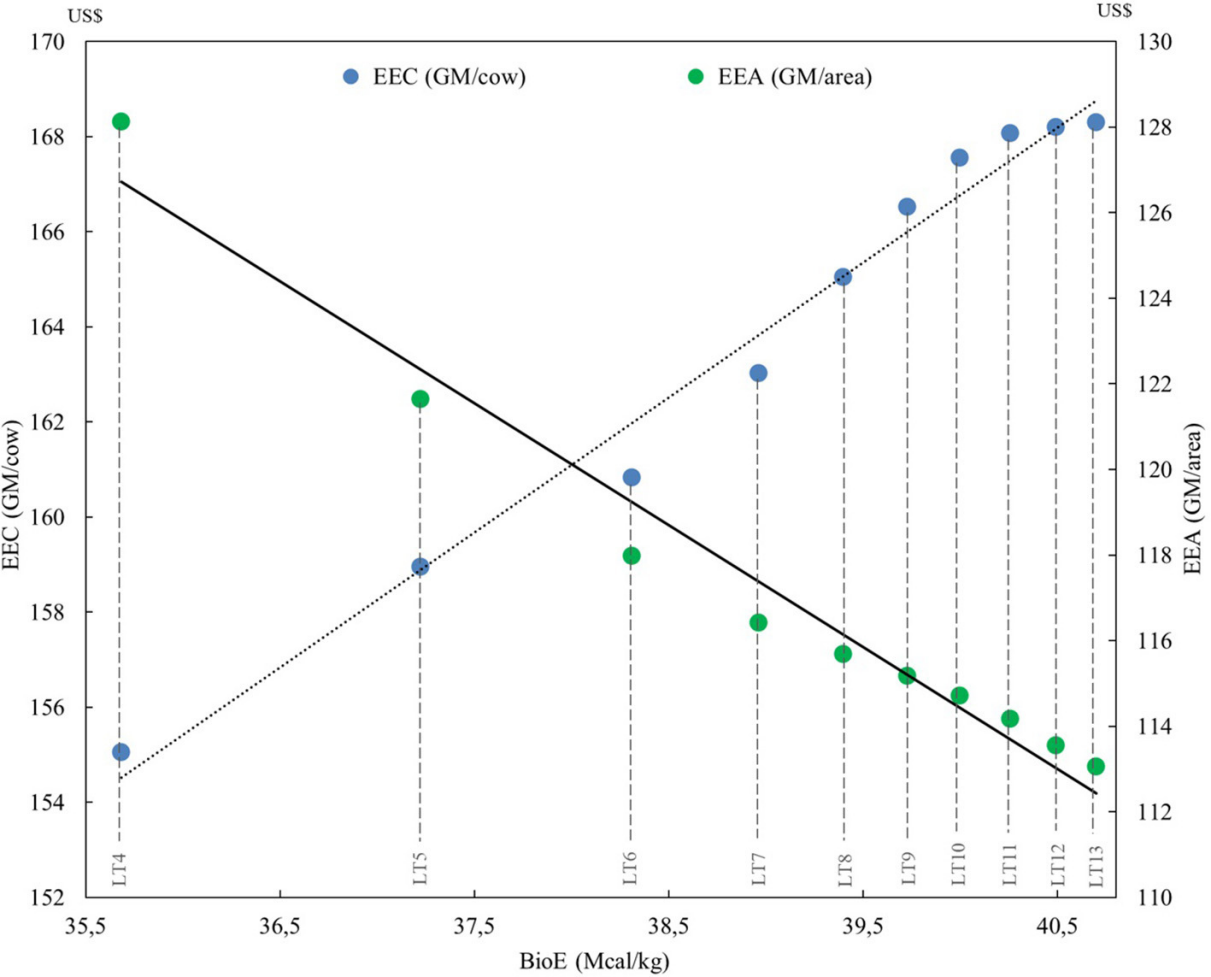
Linear regression of biological efficiency (BioE), economic efficiency per cow (EEC) (*r*^2^ = 0.96; *p* < 0.001) and economic efficiency per area (EEA) (*r*^2^ = 0.97; *p* < 0.001) of 10 cow-calf systems with different lifetime cow (LT). Crossing point of the straight lines is the optimum point between biological and economic efficiencies.

The economic efficiencies of the systems (EEC and EEA) demonstrated that EEC was directly proportional to the increase in the cow culling age (*r*^2^ = 0.96; *p* < 0.001), with the LT13 presenting an EEC 8.4% higher than the LT4, but only 2% higher than the LT8 (Figure 7). For the EEA, the results were contrary, with the superiority of the systems that culled younger cows (*r*^2^ = 0.97; *p* < 0.001). After all, among the 10 cow-calf systems, LT4 had a better EEA than LT5 (5.4%), LT6 (8.7%), and LT13 (13.4%). It is noted that the EEC obtained more regularity among the systems, since, from LT10 (US$ 161.67) to LT13 (US$ 168.40), the variation was 0.4%, while the EEA varied 1.5% between those same systems.

The higher presence of young cows in the systems also required greater investment in high-cost resources, while the presence of older cows used lower-cost resources, such as natural grasslands instead of cultivated pasture. Even with the lowest biological efficiency, the older cow systems are more economically efficient. The regression demonstrated that the bioeconomic efficiency in herds of calves that have their energy requirements met is reached when culling cows is close to 6 years. Therefore, among the simulated scenarios, LT6 was the one with the best efficiency when considering BioE, EEC, and EEA.

## Discussion

The proposed model served its purpose and was able to represent typical cow-calf systems of Southern Brazil validated by performance checks and evaluations (41). The herd structure submodel used validated experimental data and logic for its construction (1, 6, 7, 24). Our findings demonstrate coherence, maintaining the appropriate proportions between the cows' age groups in each system. In comparison with the participation of each age group of cows at calving in similar studies (1, 7), the herd structure results were similar (Table 6) and was also verified and tested to ensure credible results.

**Table 6:** Percentage of cows at calving by age groups in a cow-calf herd.

**Cow age (years)**	**2**	**3**	**4**	**5**	**6**	**7**	**8**	**9**	**10**	**11**	**12**
	**Age group of cows at calving (%)**										
Lamb et al. (1)	18	15	13	54	-						
Roberts et al. (7)	17	15	13	11	10	9	8	6	5	3	3
Model	16	14	12	11	10	8	7	7	6	5	4

*Data based on Angus cows; 87.5% average calving rate; 18% heifer replacement; 2% of cow mortality; 5% mortality of calves of 2-year-old cow; 2% mortality of calves of cows above 2 years old; 12 years of lifetime cow in the herd*.

The culling of 50% of replacement heifers after 5 years also indicates the consistency of the submodel, as similar results have been reported in researches that culled half of the heifers retained for replacement between 4 and 5 years after their insertion in the cow-calf herd (7, 42, 43). The greater number of replacement heifers in the lower LT systems is justified by the shorter lifetime cow in the herd. For the system to maintain its structure and stability, the number of replacement females must be the same as that of culled females, regardless of the reason for culling (6, 7).

Most of the equations inserted in the energy requirement submodel have been independently validated (4, 21, 32). The exposed results were also relevant, since the consumption of 8,136 Mcal of ME/cow/year in LT11 agree with the results of Lamb et al. (1).

The lower age of culling cows also increases the number of culled cows, even with the production of a calf, and the high concentration of cows in lower age groups increases the number of cows of the last calving that are culled at weaning. Despite culling a larger number of cows, the total number of animals in the herd increases, as for each culled cow, a heifer is retained for replacement. As a result, in addition to more cows from the last season from weaning to calving, there are ore heifers from weaning to the next reproductive period. Therefore, the younger culling age increases the number of animals in the herd and, consequently, these systems present a higher ECM and ME_m_.

Another factor that accentuates the energy consumption of the herd is that the lowest LT systems have the highest number of 4-year-old cows, and they have the highest MECC among all the age groups. Even if they consume less ME_m_ and ME_y_ than mature cows, they still require ME_g_ to grow 4% of their live weight (23). The ME_m_ variations in the different systems demonstrate the coherence of the results regarding energy distribution of 68% (LT4) to 72% (LT13), as established by the classic literature of 70% (3). After all, although lower LT systems consume more ME_m_, it was the higher LT systems that used most of the energy for this function, since it presents more mature animals.

These findings are essential to the rural manager who intends to reduce the culling age of cows to reduce feed consumption by the herd, believing that replacing a mature cow with a young cow will. In fact, at the beginning of the reproductive life, a young cow consumes less ME than an adult cow. However, greater cow culling will raise the system requirement overall. Therefore, it is essential that the rural manager understands the implications imposed by this change when changing the herd dynamics.

The reduction in the culling age of cow also increases the number of animals growing in the herd, since in LT4 and LT5 systems, only bulls from 5 to 7 years old have reached mature weight. The greater presence of young animals in the herd increases the ME_g_ consumption, given that this animals allocate the greatest amount of ECM for growth functions (4). So, in addition to a greater number of animals to feed, these systems have the challenge of meeting the energy requirements of more demanding cows and for this, it is necessary to invest more in better quality feed that is expensive.

The increase in production cost per cow and per system, as the LT decreases, is due to the high disbursement for cultivated pastures that have elevated implantation and maintenance costs. Nevertheless, cultivated pastures are indispensable to meet the energy requirements of low LT systems and avoid culling cows due to reproductive failures or calving difficulties (45, 46), especially for young cows. Although only 10% of the total area of LT13 consists of oat/ryegrass pasture (144 ha), this was the most representative item for this system because feed costs are among the most onerous for beef cattle production systems (47–49). Hence, it is important to clarify that the increase in the number of animals, as the LT decreases, also contributes to the increase in costs, as there is a need for a greater feed supply.

Systems that maintain cows for longer present a larger number of mature cows, due to a greater number of age groups and, consequently, less culling by age. Mature cows are the ones that most require ME_m_ and ME_y_ (4), they demand that these systems use the highest proportion of ME for these functions. The same premise is valid for ME_l_, since cows at 4 years already consume the same amount of this energy as mature cows (4). As these systems cull fewer cows by age, it is necessary to retain fewer female calves for rearing and later replacement, so the larger LT allows for selling more female calves.

It is important to highlight that this performance is only possible in systems with high weaning rates and a feed availability compatible with the herd requirements. Otherwise, all female calves will be retained for replacement, and there will be no surplus for sale. In other study in cow-calf systems with different heifer replacement rates, the herd with the least replacement showed a higher surplus of female calves for sale (7).

In contrast, the reduction in the culling age of cows in stable systems allows the increase of LW for commercialization (6). This is due to the higher average weight per animal sold in the system because of the greater proportion of cull cows sold heavier than weaned calves. It was these circumstances that allowed LT4 to achieve superiority of TLWS because, despite being the system that sold the least weaned calves, it was the one that sold the most cull cows and, consequently, obtained the highest TR. Therefore, the lower LW sale of weaned calves is offset by the higher sale of culled cows (7). In this simulation, the higher TR, due to higher productivity, is accompanied by higher production costs in systems with lower LT, as a consequence of the high system intensification to meet the energy requirements, which agrees with other *in loco* studies (49, 50).

The inverse relation between TR and LT is caused by the increase in the number of cull cows that can improve the economic results of cow-calf systems (51) without compromising the herd structure, as long as the reproductive indices are adequate. However, this strategy can damage herd structure henceforth if there is inadequate heifer replacement. In practical terms, systems with a weaning rate below 65% cannot cull all cows that failed to wean a calf. After all, in addition to the challenge of a high energy supply for growing animals and the increased production costs, the systems will not be able to replace the number of heifers needed.

Furthermore, considering that a significant number of farmers sold cows in reproductive age in past crisis, the state herds structure in the following years was also impacted, compromising the competitiveness of the entire beef supply chain (52). Moreover, the relationship between revenue and TLWS, although direct, is not proportional because the selling price of animals varies according to the category and must be considered in the selection of the best LT for the system, giving the fluctuations and market trends.

In Brazil, the slaughter of females represents, on average 41% of the cattle slaughtered in 2018 (52), while in the analyzed region the historical average is 48% (53). The main reasons for this high participation are the reproductive failures, culling age or strategies to increase the revenue of the systems, since, on average, the kg price of the cull cows is about 70% of a calf kg (38, 39), which contributes to cow-calf systems to remain competitive.

However, in other countries such as the USA, this ratio average is only 36%, with the price of cull cow kg at only 30% of calf's kg (54), which discourages farmers. Therefore, the total number of kilograms sold becomes more important than the price received, ergo, the market must be understood for the system to achieve the best economic results. In this sense, this model allows the analysis of cow-calf systems and their market to assist in decision-making regarding the configurations of the herd for better efficiency.

The best BioEC presented by the 2-year-old cow is a consequence of the lower proportion of ME for the maintenance function and the greater part for the production functions (ME_g_, ME_l_ e ME_y_) (4). Whereas, after a cow reaches 10 years, it begins a gradual weight loss (7), which also decreases the BioEC. This process of converting energy into a marketable product explains the best BioE presented by the systems with the lowest LT and confirms the hypothesis that younger cow herds are more efficient. Seidel and Whittier (6) found the best efficiency in cow-calf systems when culling cows at 2.5 years old (LT3), after the first calf. However, this system is not supported, as it is unable to produce the necessary number of replacement heifers for the next productive cycle. Therefore, the best BioE of the herd is dependent on the highest proportion of 2-year-old cows.

Although BioE was better at LT4, these systems face challenges such as high feed costs, making this LT the worst in EEC. Aside from this, the management of these herds has high operational complexity, and management failures can compromise calve production in the next cycle if the high energy requirements are not met. These results were similar to other studies, which observed a reduction in the difference in efficiency between sequential systems as the cow culling age increased. This is because there was an increase in nutrient demand and a reduction in kg production due to the greater number of mature cows (8, 12).

Because of the lower costs, GM was higher in larger LT, which also gave to LT13 the best EEC. However, the systems that culled younger cows had much smaller production areas, which gave these systems the best efficiency per area (EEA). Therefore, when considering economic efficiency from an individual point of view, the system that kept its cows longer, was more economically efficient despite being less productive and biologically efficient. Nevertheless, from the land-use point of view, those that remained less time with their cows were biologically and economically more efficient. Consequently, the system with the ideal bioeconomic efficiency is the LT6, which culled cows at about 5.5 years old.

Although this model is based on the particularities of Brazilian production, with a predominance in grassland production, the GM per cow among the systems (US$ 155.05 to US$ 168.30) is similar to countries with developed production, such as the USA, in which the average GM per cow over the past 10 years was about US$ 147.61 (55). This confirms the consistency of the model and is reliable to simulate cow-calf systems in different realities and markets, being appropriate to predict its economic results.

Additionally, even if the land costs have not been considered in this model, it is an important factor in farmers' decisions for the level of investments in the system. The increase in land prices requires production intensification through the improvement of productive indexes so that the system remains economically viable. In breeding systems, it represents an growth in productivity, as the proportion of young cows increases in the herd due to better quality forages. Therefore, expensive land is proper for young cow production to justify the high investment. In contrast, low-priced land is generally characterized by low nutritional feed or by the inadequacy for cropping, which can lead to a reduction in technological investment (56, 57). Such lands are destined for herds with a higher proportion of adult cows, which have the capacity to extract the nutrients necessary for the production of lower quality feed.

Therefore, the culling age of cows is not imperative concerning the bioeconomic efficiency of cow-calf systems, but rather a tool for decision-making, since natural resources can directly impact the herd characteristics. Aside from this, bioeconomic efficiency is dependent on the market in which the system is inserted, and this model can predict which are the best configurations or changes that rural managers should consider for this decision-making.

## Conclusion

Understanding and adjusting the lifetime cow in the herd is fundamental to the bioeconomic efficiency of cow-calf systems due to the modifications it causes in herd structure. This is because a larger number of young cows can improve the productivity and revenue of the system, but it also presents greater operational complexity and production cost than systems that keep older cows.

Even so, the shorter lifetime cow enables the increase of biological and economic efficiency when the production area is the primary resource of the system to generate the economic result. Nevertheless, the longer lifetime cow offers greater economic efficiency when the cow is the most relevant resource, even if the biological efficiency is lower. Hence, systems with younger cows are indicated for regions that allow production intensification and pay more for cull cow, while systems with older cows should be used where it is difficult to intensify and/or commercialize the cull cow profitably.

Therefore, it is not only the cow culling age that will define the best bioeconomic result of the system but also the capacity of the rural manager to understand his production system and to plan the cows' lifetime according to the opportunities that his system its capable of. Finally, the farmers also should consider and analyze the market to provide the product demanded by the customer and achieve the highest efficiency.

## Data Availability Statement

The raw data supporting the conclusions of this article will be made available by the authors, without undue reservation.

## Author Contributions

AS idealized and executed the work in all stages, from the conceptual model to the submission of the work. TO participated in the textual construction. FL-G assisted in the development of the model. DF participated in the construction of the method and results. JB guided and reviewed the study from start to finish. All authors contributed to the article and approved the submitted version.

## Conflict of Interest

The authors declare that the research was conducted in the absence of any commercial or financial relationships that could be construed as a potential conflict of interest.

## References

[1] Lamb MA Tess MW Robison OW Evaluation of mating systems involving five breeds for integrated beef production systems: I. Cow-calf segment. J Anim Sci. (1992) 70:689–99. 10.2527/1992.703689x 1563995

[2] Walmsley BJ Lee SJ Parnell PF Pitchford WS A review of factors influencing key biological components of maternal productivity in temperate beef cattle. Anim Prod Sci. (2016) 58:1–19. 10.1071/AN12428

[3] Ferrell CL Jenkins TG. Cow type and the nutritional environment: nutritional aspects. J Anim Sci. (1985) 61:725–41. 10.2527/jas1985.613725x 4066531

[4] National Research Council (NRC). Nutritional Requirements of Beef Cattle. Update (2000), Seventh Revised Edition. Washington, DC: National Academy Press (1996).

[5] Brethour JR Jaeger JR. The single-calf heifer system. Kansas Agric. (1989). 570.

[6] Seidel JGE Whittier JC. Beef species symposium: Beef production without mature cows. J Anim Sci. (2015) 93:4244–51. 10.2527/jas.2014-8526 26440323

[7] Roberts AJ Petersen MK Funston RN. Can we build the cowherd by increasing longevity of females? J Anim Sci. (2015) 93:4235–43. 10.2527/jas.2014-8811 26440322

[8] Taylor CS Moore AJ Thiessen RB Bailey CM Efficiency of food utilization in traditional and sex-controlled systems of beef production. Anim Prod. (1985) 40:401–40. 10.1017/S0003356100040125

[9] Bourdon RM Brinks JS. Simulated efficiency of range beef production. III culling strategies and nontraditional management systems. J Anim Sci. (1987) 65:963–9. 10.2527/jas1987.654963x 3667469

[10] Ereth BA Whittier JC Burns PD Schultz DN Couch DW Seidel GE Integration of early weaning and sexed semen into a single-calf heifer system to increase value of non-replacement heifers. Proc Am Soc Anim Sci. (2000) 51:441–3.

[11] Rotz CA Isenberg BJ Stackhouse-Lawson KR Pollak EJ. A simulation-based approach for evaluating and comparing the environmental footprints of beef production systems. J Anim Sci. (2013) 91:5427–37. 10.2527/jas.2013-6506 24146148

[12] Naazie A Makarechian M Hudson RJ. Evaluation of life-cycle herd efficiency in cow-calf systems of beef production. J Anim Sci. (1999) 77:1–11. 10.2527/1999.7711 10064021

[13] Feuz DM Skold MD Typical farm theory in agricultural research. J Sustain Agric. (1990) 2:43–58. 10.1300/J064v02n02_05

[14] Rumpf JM Van Vleck LD. Age-of-dam adjustment factors for birth and weaning weight records of beef cattle: a review. Genet Mol Res. (2004) 3:1–17. Available online at: https://www.geneticsmr.com/sites/default/files/articles/year2004/vol3-1/pdf/gmr0089.pdf 15100984

[15] Gregory KE Echtemkamp ES Dickerson GE Cundiff LV Koch RM Van Vleck LD. Twinning in cattle: III. Effects of twinning on dystocia, reproductive traits, calf survival, calf growth and cow productivity. J Anim Sci. (1990) 68:3133–44. 10.2527/1990.68103133x 2254192

[16] Hansen PJ Baik DH Rutledge JJ Hauser ER. Genotype X environmental interactions on reproductive traits of bovine females. II Postpartum reproduction as influenced by genotype, dietary regimen, level of milk production and parity. J Anim Sci. (1982) 55:1458–72. 10.2527/jas1982.5561458x 7161217

[17] Butson S Berg RT Lactation performance of range beef and dairy-beef cows. Can J Anim Sci. (1984) 64:253–65. 10.4141/cjas84-032

[18] Butson S Berg RT Factors influencing lactation performance of range beef and dairy-beef cows. Can J Anim Sci. (1984) 64:267–77. 10.4141/cjas84-033

[19] Clutter AC Nielsen MK. Effect of level of beef cow milk production on pre-and postweaning calf growth. J Anim Sci. (1987) 64:1313–22. 10.2527/jas1987.6451313x 3583941

[20] Rovira J Manejo nutritivo de los rodeos de cria em pastoreo. Montevideo: Hemisferio Sur (1996). p. 288.

[21] Agricultural and Food Research Council (AFRC) Energy and Protein Requirements of Ruminants. Wallingford: Commonwealth Agricultural Bureaux International (1993).

[22] Gregory KE Cundiff LV Koch RM. Breed effects and heterosis in advanced generations of composite populations on actual weight, adjusted weight, hip height, and condition score of beef cows. J Anim Sci. (1992) 70:1742–54. 10.2527/1992.7061742x 1634398

[23] Fox DG Sniffen CJ O'Connor JD Russell JB Van Soest PJ. A net carbohydrate and protein system for evaluating cattle diets: III. Cattle requirements and diet adequacy. J Anim Sci. (1992) 70:3578–96. 10.2527/1992.70113578x 1334063

[24] Pang H Makarechian MH Basarab JA Berg RT Structure of a dynamic simulation model for beef cattle production systems. Can J Anim Sci. (1999) 79:409–17. 10.4141/A99-020

[25] Houghton PL Lemenager RP Moss GE Hendrix KS Prediction of postpartum beef cow body composition using weight to height ratio and visual body condition score. J Anim Sci. (1990) 68:1428–37. 10.2527/1990.6851438x

[26] López-González FA Allende R Lima JMS Canozzi MEA Sessim AG Barcellos JOJ Intensification of cow-calf production: how does the system respond biologically to energy inputs in a long-term horizon? Livest Sci. (2020) 237:104058 10.1016/j.livsci.2020.104058

[27] Dunn TG Ingalls JE Zimerman DR Wiltbank JN. Reproductive performance of 2-year-old Hereford and Angus heifers as influenced by pre-and post-calving energy intake. J Anim Sci. (1969) 29:719–26. 10.2527/jas1969.295719x 5391970

[28] Goehring TB Corah LR Higgins JJ. Effects of energy and lasalocid on productivity of first-calf heifers. J Anim Sci. (1989) 67:1879–88. 10.2527/jas1989.6781879x 2793617

[29] Dunne LD Diskin MG Sreenan JM. Embryo and foetal loss in beef heifers between day 14 of gestation and full term. Anim Reprod Sci. (2000) 58:39–44. 10.1016/S0378-4320(99)00088-3 10700643

[30] Diskin MG Morris DG. Embryonic and early foetal losses in cattle and other ruminants. Reprod Dom Anim. (2008) 43:260–7. 10.1111/j.1439-0531.2008.01171.x 18638133

[31] Nasca J Feldkamp CR Arroquy JI Colombatto D Efficiency and stability in subtropical beef cattle grazing systems in the northwest of Argentina. Agric Syst. (2015) 133:85–96. 10.1016/j.agsy.2014.10.014

[32] National Research Council (NRC) Nutritional Requirements of Beef Cattle. Eighth Revised Edition. Washington, DC: National Academy Press (2016).

[33] Restle J Roso C Soares AB Produção animal e retorno econômico em misturas de gramíneas anuais de estação fria. Rev Bras de Zoot. (1999) 28:235–43. 10.1590/S1516-35981999000200003

[34] Roso C Restle J Soares AB Alves Filho D Brondani IL Produção e qualidade de forragem da mistura de gramíneas anuais de estação fria sob pastejo contínuo. Rev Bras de Zoot. (1999) 28:459–67. 10.1590/S1516-35981999000300004

[35] Piazzetta RG Dittrich JR Alves SJ Moraes AD Lustosa SBC Gazda TL Características qualitativas da pastagem de aveia preta e azevém manejada sob diferentes alturas, obtida por simulação de pastejo. Arch Vet Sci. (2009) 14:43–8. 10.5380/avs.v14i1.12636

[36] Silveira EO Produção e comportamento ingestivo de cordeiros em pastagem de azevém anual (Lolium multiflorum Lam.) manejada em diferentes alturas (dissertação de Mestrado em Zootecnia), Faculdade de Agronomia, Universidade Federal do Rio Grande do Sul, Porto Alegre, Rio Grande do Sul (2001).

[37] Carvalho PCF Wallau MO Bremm C Bonnet O Trindade JK da Rosa FQ Nativão: 30 anos de pesquisa em campo nativo. Porto Alegre: Universidade Federal do Rio Grande do Sul, Faculdade de Agronomia, Departamento de Plantas Forrageiras e Agrometeorologia (2017) 146.

[38] ESALQ-CEPEA Indicador do bezerro esalq/bmandfbovespa – Data Base. (2019). Available online at: https://www.cepea.esalq.usp.br/br/consultas-ao-banco-de-dados-do-site.aspx (accessed May 20, 2019).

[39] Núcleo de Estudos em Sistemas de Produção de Bovinos de Corte e Cadeia Produtiva (NESPro) NESPro Indices - Data Base. (2019) Available online at: http://www.ufrgs.br/nespro/nespro_indices_categorias_bovinos.php (accessed October 27, 2019).

[40] IBM Corp Released. IBM SPSS Statistics for Windows, Version 20.0. Armonk, NY: IBM Corp (2015).

[41] Dent JB Blackie MJ Model-evaluation. In: Systems Simulation in Agriculture. Dordrecht: Springer (1979). p. 94–117. 10.1007/978-94-011-6373-6_5

[42] Schons D Hohenboken WD Hall JD Population analysis of a commercial beef cattle herd. J Anim Sci. (1985) 61:44–54. 10.2527/jas1985.61144x

[43] Tanida H Hohenboken WD DeNise SK. Genetic aspects of longevity in angus and hereford cows. J Anim Sci. (1988) 66:640–7. 10.2527/jas1988.663640x 3378921

[44] Tozer PR Scollard DL Marsh TL Marsh TJ Recursive systems model of fetal birth weight and calving difficulty in beef heifers. Can J Anim Sci. (2002) 82:19–27. 10.4141/A01-028

[45] Rogers PL Gaskins CT Johnson KA MacNeil MD. Evaluating longevity of composite beef females using survival analysis techniques. J Anim Sci. (2004) 82:860–6. 10.2527/2004.823860x 15032444

[46] Stockton MC Wilson RK Feuz DM Stalker LA Funston RN Bioeconomic factors of beef heifer maturity to consider when establishing criteria to optimally select and/or retain herd replacements. J Anim Sci. (2014) 92:4733–40. 10.2527/jas.2014-8010 25149330

[47] Bouquet A Fouilloux MN Renand G Phocas F Genetic parameters for growth, muscularity, feed efficiency and carcass traits of young beef bulls. Livest Sci. (2010) 129:38–48. 10.1016/j.livsci.2009.12.010

[48] Åby BA Aass L Sehested E Vangen O Effects of changes in external production conditions on economic values of traits in Continental and British beef cattle breeds. Livest Sci. (2012) 150:80–93. 10.1016/j.livsci.2012.08.002

[49] Åby BA Aass L Sehested E Vangen O A bio-economic model for calculating economic values of traits for intensive and extensive beef cattle breeds. Livest Sci. (2012) 143:259–69. 10.1016/j.livsci.2011.10.003

[50] Anderson VL Ilse BR Engel CL Drylot vs. pasture beef cow/calf production: three-year progress report. Beef Cattle and Range Research Report. (2013) 95:13–6. Available online at: https://www.ag.ndsu.edu/livestockextension/research-reports/2013-beef-cattle-and-range-research-report/drylot-vs.-pasture-beef-cow-calf-production-three-year-progress-report/view

[51] Turner BL Rhoades RD Tedeschi LO Hanagriff RD McCuistion KC Dunn BH Analyzing ranch profitability from varying cow sales and heifer replacement rates for beef cow-calf production using system dynamics. Agric Syst. (2013) 114:6–14. 10.1016/j.agsy.2012.07.009

[52] Instituto Brasileiro de Geografia e Estatística (IBGE) Indicadores IBGE – Estatística da Produção Pecuária. Rio de Janeiro, Vol. 52 (2018).

[53] Núcleo de Estudos em Sistemas de Produção de Bovinos de Corte e Cadeia Produtiva (NESPro) Informativo NESPro - Data Base. (2018). Available online at: http://www.ufrgs.br/nespro/informativos/4/mobile/index.html#p=2 (accessed Oct 27, 2019).

[54] United States Department of Agriculture (USDA) US Red Meat and Poultry Forecasts – Economic Research Service - Data Base. (2019). Available online at: https://usda.library.cornell.edu/concern/publications/g445cd121?locale=en (accessed 27 October, 2019).

[55] United States Department of Agriculture (USDA) Historical Costs and Returns: Cow-Calf - Data Base. (2019). Available online at: https://www.ers.usda.gov/data-products/commodity-costs-and-returns/commodity-costs-and-eturns/#Historical%20Costs%20and%20Returns:%20Cow-Calf (accessed October 31, 2019).

[56] Barros GSC ZEN S Bacchi MRP Ichihara SM Osaki M Ponchio LA Economia da Pecuária de Corte na Região Norte do Brasil. Piracicaba: Centro de Estudos Avancados em Economi Aplicada (CEPEA)-ESALQ/USP (2002). p. 76.

[57] Lampert VN Barcellos JOJ Neto FJK Canellas LC Dill MD Canozzi MEA Development and application of a bioeconomic efficiency index for beef cattle production in Rio Grande do Sul, Brazil. Rev Bras Zoot. (2012) 41:775–82. 10.1590/S1516-35982012000300042

